# ABA receptor isoforms differently regulate stomatal movements and generation of reactive oxygen species in ABA signaling in Arabidopsis guard cells

**DOI:** 10.1093/pcp/pcaf102

**Published:** 2025-08-28

**Authors:** Ye Yin, Yuki Hayashi, Monira Sirajam, Oumayma Shaiek, Shintaro Munemasa, Yoshimasa Nakamura, Toshinori Kinoshita, Yoshiyuki Murata, Izumi C Mori

**Affiliations:** Graduate School of Environmental and Life Science, Okayama University, 1-1-1Tsushima-naka, Kita-ku, Okayama 700-8530, Japan; College of Horticulture, Qingdao Agricultural University, 700 Chengyang, Chengyang, Qingdao 266109, China; Graduate School of Science, Nagoya University, Furo-cho, Chikusa, Nagoya 464-8602, Japan; Graduate School of Environmental and Life Science, Okayama University, 1-1-1Tsushima-naka, Kita-ku, Okayama 700-8530, Japan; Graduate School of Environmental and Life Science, Okayama University, 1-1-1Tsushima-naka, Kita-ku, Okayama 700-8530, Japan; Graduate School of Environmental and Life Science, Okayama University, 1-1-1Tsushima-naka, Kita-ku, Okayama 700-8530, Japan; Graduate School of Environmental and Life Science, Okayama University, 1-1-1Tsushima-naka, Kita-ku, Okayama 700-8530, Japan; Graduate School of Science, Nagoya University, Furo-cho, Chikusa, Nagoya 464-8602, Japan; Institute of Transformative Bio-Molecules (WPI-ITbM), Nagoya University, Furo-cho, Chikusa, Nagoya 464-8602, Japan; Graduate School of Environmental and Life Science, Okayama University, 1-1-1Tsushima-naka, Kita-ku, Okayama 700-8530, Japan; Institute of Plant Science and Resources, Okayama University, 2-20-1chuo, Kurashiki, Okayama 710-0046, Japan

**Keywords:** abscisic acid, pyrabactin, ABA receptors, reactive oxygen species, stomatal closure, stomatal opening

## Abstract

ABA signaling in stomatal guard cells is crucial for plants to cope with abiotic stress condition. Pyrabactin is a synthetic agonist of ABA that has a selective affinity to limited isoforms of ABA receptors. Here, we investigated the differential utilization of downstream signaling events in guard cell ABA signaling under specific receptor isoforms taking advantage of pyrabactin affinity. Pyrabactin-induced stomatal closure as well as ABA, while it did not inhibit stomatal opening. Plasma membrane inwardly rectifying K^+^ channel was not regulated by pyrabactin, while H^+^-ATPase activation was negatively regulated by pyrabactin. Pharmacological and molecular genetic evidence supported that reactive oxygen species production occurred differentially between the closure-inducing and opening-inhibiting signals in guard cells. These findings offer a previously unidentified mechanism for ABA signaling events promoting closure induction and opening inhibition of stomata, which were distinct from each other and governed by different ABA receptor isoforms discriminable by their affinity for pyrabactin.

## Introduction

Stomata are tiny pores surrounded by a pair of guard cells on the epidermis in the aerial parts of plants and play a crucial role in CO_2_ uptake and water loss (Hetherington and Woodward 2003, Murata et al. 2015). ABA is a natural plant hormone that plays a key role in regulating stomatal movement (Schroeder et al. 2001). ABA exerts its effects by binding to specific receptor isoforms, the PYRABACTIN RESISTANCE 1 (PYR1)/PYR1-LIKE (PYL)/REGULATORY COMPONENT OF ABA RECEPTOR (RCAR) protein family (hereafter called PYLs), which consists of 14 members in the Arabidopsis genome (Ma et al. 2009, Park et al. 2009).

It was reported that ABA-induced gene expression in guard cells was differentially regulated under different ABA receptor isoforms (Dittrich et al. 2019). Not only the regulation of gene expression but also opening inhibition and closure induction of stomata are differentially regulated under different ABA receptors (Yin et al. 2013, Assmann and Jegla 2016). These reports strongly support the notion that each ABA receptor isoform carries out a different function. However, the roles of each ABA receptor isoform have not been well elucidated.

Pyrabactin is a synthetic ABA agonist and has a selective affinity among ABA receptor isoforms (Park et al. 2009, Hao et al. 2010). Reportedly, a subset of dimeric ABA receptors, PYR1 and PYL1, have a high affinity for binding to pyrabactin; one of the monomeric ABA receptors, PYL10, has a low affinity to pyrabactin; and the other ABA receptors have a negligible affinity to it in Arabidopsis (Melcher et al. 2010, Okamoto et al. 2013). It was shown that the gene expression regulated by pyrabactin and ABA is similar in seeds. In seedlings, however, a poorer correlation in the gene expression profile was noticed (Park et al. 2009). These expression profile differences may be explained by the distinct participation of ABA receptor isoforms in each tissue. Referring to stomatal aperture regulation, little is known about differences in functions among PYL isoforms. One of the aims of this study is to explore the difference in the roles of ABA receptor isoforms in stomatal movement and ABA signaling processes in guard cells using pyrabactin.

Reactive oxygen species (ROS) generated by NADPH oxidases are key signaling molecules in the regulation of stomatal movements in response to environmental stimuli (Pei et al. 2000, Zhang et al., 2001b, Kwak et al. 2003, Sirichandra et al. 2009). H_2_O_2_ is a relatively stable ROS conveying spatiotemporal signals in guard cells (Mori and Schroeder 2004). H_2_O_2_ inhibits inwardly rectifying K^+^ (K_in_) channels, activates hyperpolarization-activated Ca^2+^-permeable (*I*_Ca_) channels, and eventually induces stomatal closure (Pei et al. 2000, Zhang et al., 2001a). H_2_O_2_ was also reported to be involved in ABA-induced dephosphorylation of H^+^-translocating adenosine 5′-triphosphatase (H^+^-ATPase). Additionally, it facilitates the inhibition of stomatal opening by ABA as well as the induction of closure (Zhang et al. 2004, Yan et al. 2007). On the other hand, it was reported that the manner of ROS production was diversified. The inducible cytosolic H_2_O_2_ elevation contributes to ABA-induced stomatal closure, while the constitutive increase of H_2_O_2_ does not cause stomatal closure (Jannat et al. 2011). A previous study suggested that cytosolic H_2_O_2_ accumulation was a prerequisite for stomatal closure induction (Yin et al. 2013). In that study, it was claimed that cytosolic H_2_O_2_ accumulation was not involved in opening inhibition based on the absence of a gain in fluorescence of a cytosolic H_2_O_2_ indicator in guard cells (Yin et al. 2013). However, this statement remains to be further elucidated since the involvement of other ROS besides H_2_O_2_ in the cytosol is potentially conceivable. This study also aims to re-examine the roles of ROS in inhibiting stomatal opening during the ABA response of guard cells regulated under specific ABA receptor isoforms.

## Results

### Pyrabactin does not inhibit light-induced stomatal opening, while ABA does

Some ABA receptors possess a high affinity to the ABA agonist, pyrabactin (Melcher et al. 2010, Okamoto et al. 2013). Taking advantage of its unique affinity, we examined pyrabactin on two distinct stomatal responses: closure induction and opening inhibition, to elucidate the differential contribution of ABA receptor isoforms.

**Figure 1:**
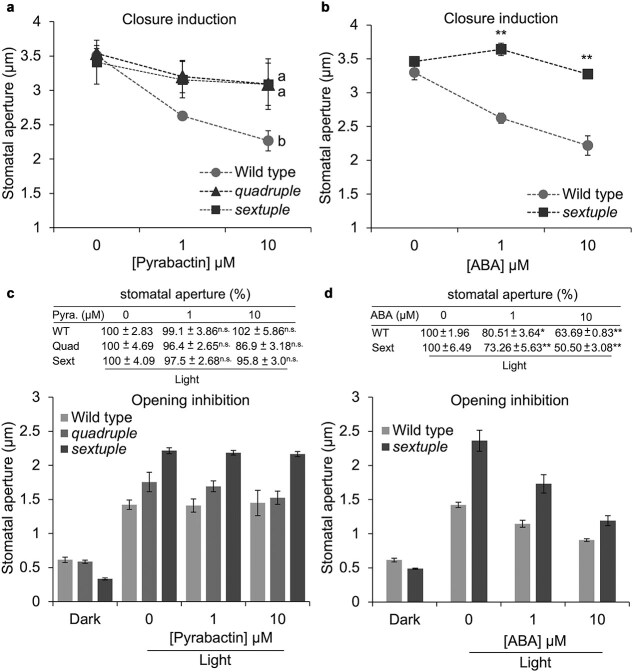
Partly different effects of ABA and the ABA agonist, pyrabactin, on stomatal movements. (a) Pyrabactin-induced stomatal closure in wild type, ABA receptor mutants, *quadruple* (*pyr1 pyl1 pyl2 pyl4*), and *sextuple* (*pyr1 pyl1 pyl2 pyl4 pyl5 pyl8*). Same letters indicate not significant at 5% level by Tukey’s honestly significant difference test. (b) ABA-induced stomatal closure in wild type and sextuple mutant. Asterisks indicate a significant difference between wild type and sextuple mutant at 5% significance level by Student’s *t*-test. (c) Effect of pyrabactin on stomatal opening in wild type, quadruple mutant, and sextuple mutant. The table at the top indicates aperture widths are indicated as % normalized to that of the solvent control (0.1% DMSO, 0 μM pyrabactin). The mean values of stomatal aperture widths are shown in bar graph together with the results in the dark condition. (d) Effect of ABA on stomatal opening in wild type and sextuple mutant. The table at the top indicates aperture widths are indicated as %, which are normalized to that of the solvent control (0.1% ethanol, 0 μM ABA). The mean values of stomatal aperture widths were shown in bar graph together with the results in the dark condition. (c and d) Asterisks indicate significant differences from each control at 5% significance level (*) or 1% significance level (**) by Dunnett’s test. n.s., not significant. Averages from five independent experiments are shown (20 stomata were measured per experiment). Error bars represent SE of the mean.

Application of exogenous pyrabactin to the excised leaf of wild-type Arabidopsis induced stomatal closure as well as application of ABA (Fig. 1a and b) in agreement with the earlier study (Okamoto et al. 2013). An incubation of dark-adapted purified epidermis in the light condition induced stomatal opening in the absence of pyrabactin from 0.61 ± 0.04 μm to 1.42 ± 0.07 μm. The opening was not inhibited in the presence of 1 and 10 μM pyrabactin (Fig. 1c) in contrast to ABA (Fig. 1d).

In addition to wild type, the effects of ABA and pyrabactin on stomatal movement were examined in two ABA receptor mutants, *pyr1pyl1pyl2pyl4* quadruple (Park et al. 2009) and *pyr1 pyl1 pyl2 pyl4 pyl5 pyl8* sextuple mutants (Gonzalez-Guzman et al. 2012). These mutants have a defect in ABA-induced stomatal closure (Yin et al. 2013 and Supplementary Table S1; Gonzalez-Guzman et al. 2012 and Fig. 1b). Pyrabactin did not induce stomatal closure in either mutant (Fig. 1a) in contrast to the wild-type plants. Pyrabactin also did not inhibit the light-induced stomatal opening in the quadruple and sextuple mutants (Fig. 1c) as well as in the wild type. It is noticeable that opening inhibition by ABA occurred more intensely in the sextuple mutant compared with the wild type (Fig. 1d), most likely due to the wider opening of the sextuple mutant’s stomata in the control condition.

These results indicate that pyrabactin in the regulation of stomatal movements is partly distinct from that of ABA. The phenotype of the sextuple mutant suggests that pyrabactin-binding ABA receptors are not sufficient for opening inhibition.

### Regulation of *I*_Kin_ by pyrabactin

**Figure 2:**
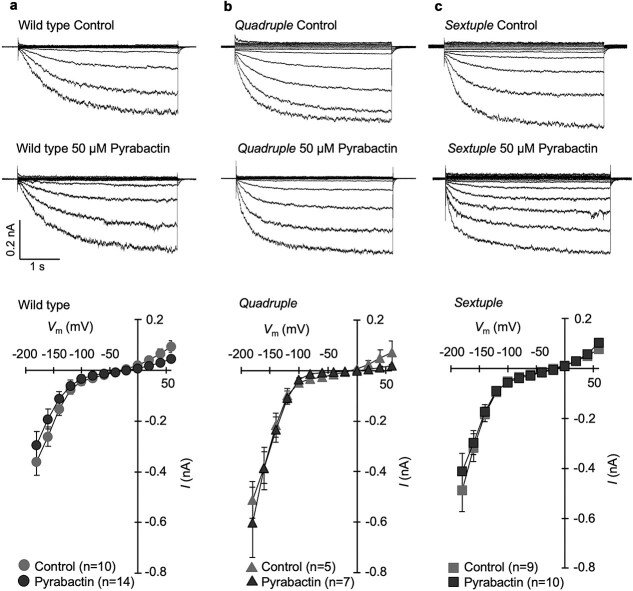
Effect of pyrabactin on *I*_Kin_ in guard-cell protoplasts. (a) Wild type. (b) *Quadruple* mutant (*pyr1 pyl1 pyl2 pyl4*). (c) *Sextuple* mutant (*pyr1 pyl1 pyl2 pyl4 pyl5 pyl8*). Top panels indicate typical raw traces of *I*_Kin_ in the absence (above) and presence of 50 μM pyrabactin (below). Bottom panels indicate current–voltage curve at steady-state current (3–3.5 s). Error bars indicate SE of the mean. *I* and *V*_m_ indicate whole-cell currents and membrane voltage, respectively.

Transport of K^+^ across the guard cell plasma membrane is critical for stomatal movement. Earlier studies have shown that ABA inhibits *I*_Kin_ in guard cells (Roelfsema and Hedrich 2005, Jezek and Blatt 2017). Here, we investigated the effect of pyrabactin on the amplitude of *I*_Kin_ in isolated Arabidopsis guard cell protoplasts by the whole-cell patch clamping (Fig. 2). The application of 50 μM pyrabactin did not inhibit *I*_Kin_ in the wild type (Fig. 2a) in contrast to 50 μM ABA (Yin et al. 2013). As shown in Fig. 2b and c, the quadruple and sextuple mutants also demonstrated the lack of inhibitory effect of pyrabactin on *I*_Kin_. It is noted that the current amplitude was larger in these mutants than in wild type. This may be explained by the effect of ABA to reduce the expression of *KAT1* and *AKT1*, K^+^ channel genes in guard cells (Inoue et al. 2017). This result suggests that pyrabactin does not regulate the inactivation of *I*_Kin._

### Pyrabactin inhibits blue light-induced phosphorylation of plasma membrane H^+^-ATPase

Activation of H^+^-ATPase is a key step in light-induced stomatal opening (Shimazaki et al. 1986). Phosphorylation of the penultimate threonine residue of H^+^-ATPase is the hallmark of the H^+^-ATPase activation (Kinoshita and Shimazaki 1999).

**Figure 3:**
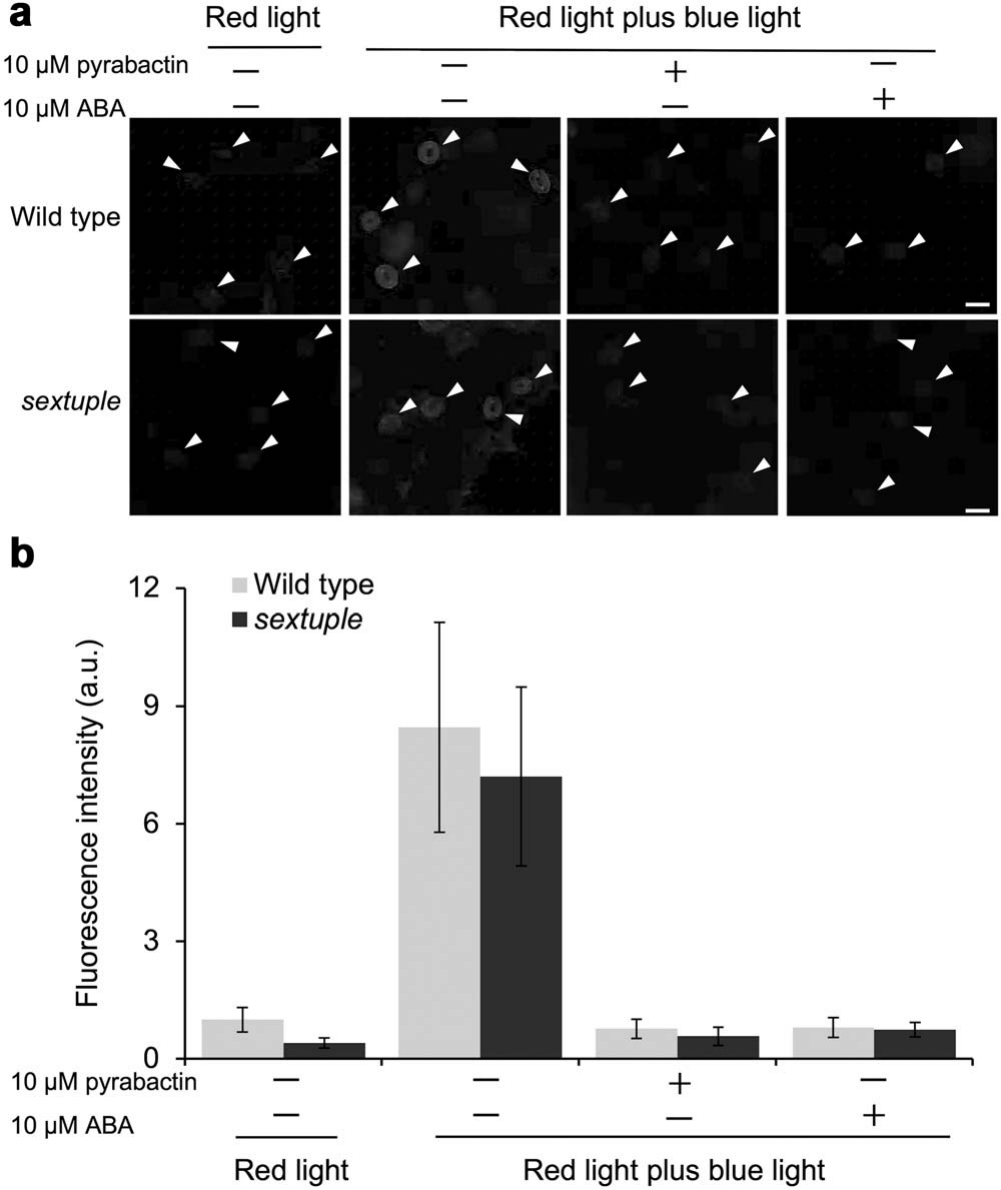
Inhibition of blue light-induced phosphorylation of plasma membrane H^+^-ATPase by pyrabactin and ABA. (a) Typical fluorescence images of stomata labeled with anti-pThr antiserum. Scale bar: 20 μm. Arrowheads indicate the position of stomata. (b) Semi-quantification of fluorescence intensity in guard cells. Epidermal fragments illuminated with background red light for 20 min were successively illuminated with red light plus blue light for 2.5 min as described in materials and methods. Where indicated, pyrabactin or ABA was added before the illumination of background red light. *n* = 3 independent experiments. One hundred twenty guard cells were measured in each experiment. No significant difference between wild type and sextuple mutant was found at 5% significance level by Student’s *t*-test in all four conditions. Error bars represent SE of the mean.

The effect of pyrabactin on phosphorylation of the penultimate threonine in the plasma membrane H^+^-ATPase in guard cells was examined by immunohistochemical staining using a specific antiserum (Hayashi et al. 2011, Yin et al. 2013). In the background red light, H^+^-ATPases were slightly phosphorylated in wild type and sextuple mutant guard cells (Fig. 3). Illumination with blue light superimposed on the background red light augmented the phosphorylation of H^+^-ATPase in both wild type and the sextuple mutant (Fig. 3). Addition of 10 μM pyrabactin completely inhibited the phosphorylation of H^+^-ATPase in the wild type and sextuple mutant like ABA (Fig. 3). It is noticeable that stomatal opening was not suppressed by pyrabactin, while the activation of H^+^-ATPase was inhibited by pyrabactin. These results indicate that ABA receptors PYR1, PYL1, PYL2, PYL4, PYL5, and PYL8 are not sufficient for the signaling process to inhibit H^+^- ATPase activation and suggest that some of other PYLs members or unidentified ABA receptor(s) that can bind to pyrabactin are involved in the deactivation of H^+^-ATPase. Although it was established that the inactivation of H^+^-pump is essential for opening inhibition (Shimazaki et al. 1986, Takemiya et al. 2006), our result showed that the activation of H^+^-ATPase is not exclusively satisfactory for opening induction.

### Regulation of second messenger mobilization by pyrabactin

**Figure 4:**
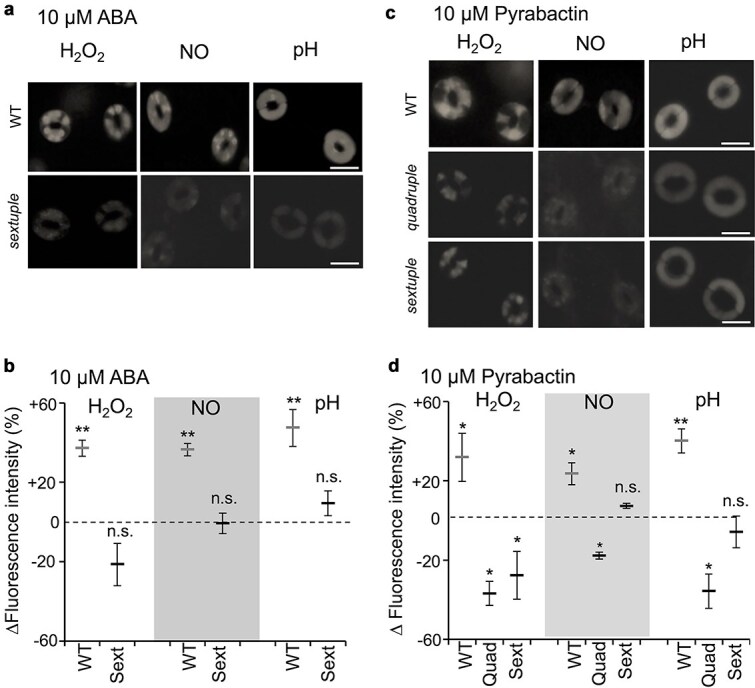
Up- and downregulation of H_2_O_2_ and NO accumulation levels and cytosolic pH by pyrabactin in quadruple and sextuple mutant guard cells. (a and c) representative fluorescence images of H_2_DCF-stained (H_2_O_2_), DAF-stained (NO), and BCECF-stained (pH) guard cells in the presence of ABA or pyrabactin. Scale bar = 10 μm. (b and d) fluorescence intensity difference relative to the control conditions (dotted horizontal line) was shown as mean ± SE (3 biological replicates, 50 guard cells per replicate). WT, wild type. Quad, *pyr1 pyl1 pyl2 pyl4* quadruple mutant. Sext, *pyr1 pyl1 pyl2 pyl4 pyl5 pyl8* sextuple mutant. Asterisks indicate significant difference at 5% significance level (*) or 1% significance level (**) by Student’s *t*-test from each control. n.s., not significant.

ROS production, nitric oxide (NO) production and cytosolic alkalization are postulated to act as second messengers in ABA signaling in guard cells (Irving et al. 1992, Pei et al. 2000, Desikan et al. 2002). Reportedly, ABA failed to induce ROS production, NO production, and cytosolic alkalization in the quadruple mutant (Yin et al. 2013 and Supplementary Table S2). In the sextuple mutant, the mobilization of these second messengers by ABA treatment did not occur, too (Fig. 4a and b). Results so far showed that H_2_O_2_ production occurred concomitantly with NO production and cytosolic alkalization, suggesting a close association of these second messenger mobilizations in ABA signaling. Here, we addressed potentiality of differential regulations of these second messengers in ABA signaling using pyrabactin and ABA receptor mutants. Pyrabactin induced an increase in fluorescence levels of the H_2_O_2_ indicator, dichlorodihydrofluorescein diacetate (H_2_DCF-DA); NO indicator, diaminofluorescein-2 diacetate (DAF-2DA); and pH indicator, 2′,7′-bis (carboxyethyl)-5,6-carboxyfluorescein acetomethylester (BCECF-AM), by 31 ± 10%, 24 ± 5.5% and 40 ± 6%, respectively, in wild type guard cells (Fig. 4c and d). We initially anticipated from these results that levels of H_2_O_2_, NO, and pH in mutants’ guard cells treated with pyrabactin would stay similar to those in the control treatment. However, H_2_O_2_, NO, and alkalization levels were significantly reduced to −37 ± 6%, −18 ± 1.8% and −36 ± 8%, respectively, in the quadruple mutant (Fig. 4d). Similarly, the H_2_O_2_ levels in the sextuple mutant decreased significantly to −28% ± 10% (Fig. 4d). Interestingly, however, NO and alkalization levels stayed similar to the control treatment in the sextuple mutant (Fig. 4d). We questioned whether these downregulations of the second messenger mobilizations by ABA treatment had occurred in the quadruple mutant in the previous study (Yin et al. 2013). To address this, we examined the data. A slight decrease in the levels of fluorescent indicators by 10 μM ABA occurred superficially in the quadruple mutant’s guard cells (Supplementary Table S2). However, a statistical analysis revealed that *P*-values for the reduction of fluorescence [98.4 ± 3.1% (mean ± SD) for H_2_DCF and 95.6 ± 3.5% for DAF] were higher than 0.05 (*P* = .2 and .11 by Student’s *t*-test, respectively). On the other hand, the BCECF fluorescence was reduced statistically significantly (90.6 ± 1.7%, *P* = .03).

These results indicate that pyrabactin has partly different actions from ABA on mobilization and feedback down-regulations of second messengers in guard cells, which become observable in the mutant background (Fig. 4). Perhaps, distinct sets of ABA receptors regulate the activation of ABA signaling accompanying feedback down-regulations of second messengers in an intricate manner.

### Involvement of ROS generation in opening inhibition

ROS production, NO production, and cytosolic alkalization responses showed trivial differences between ABA and pyrabactin (Fig. 4). Meanwhile, ABA and pyrabactin effects on opening inhibition were clearly distinct (Fig. 1a). These complicated results led us to investigate the involvement of ROS in opening inhibition in further detail. Here, we took molecular genetic and pharmacological approaches to examine the involvement of ROS generation in the inhibition of light-induced stomatal opening. DPI is an inhibitor of flavoproteins including NADPH oxidase. Tiron (1,2-dihydroxybenzene-3,5-disulfonate) and *N*-acetylcysteine (NAC) can act as chemical scavengers for various biologically relevant oxidants with different substrate specificities. The reaction constant of tiron for O_2_^−^• is very high (5 × 10^8^ M^−1^ s^−1^) (Greenstock et al. 1975). On the other hand, that of NAC for O_2_^−^• is low (6.8 × 10^−1^ M^−1^ s^−1^), while that for OH• is extremely high (>10^10^ M^−1^ s^−1^) (Aruoma et al. 1989, Aldini et al. 2018).

Stomatal closure induced by ABA and pyrabactin was impaired in the NADPH oxidase double knockout mutant, *atrbohD/F* (Supplementary Figs S1a and S2a). DPI also strongly inhibited stomatal closure (Supplementary Fig. S1b). To further ascertain the participation of ROS in the closure induction process, the effects of ROS scavenging agents, tiron and NAC were examined. Tiron partially but significantly inhibited ABA-induced stomatal closure (Supplementary Fig. S1c). Notably, it was not inhibited or was modestly inhibited by NAC in wild type (Supplementary Fig. S1d). Inferring the specificity of the scavengers to ROS, the involvement of O_2_^−^• in closure induction is strongly suggested, while OH• is not or is less involved.

**Figure 5:**
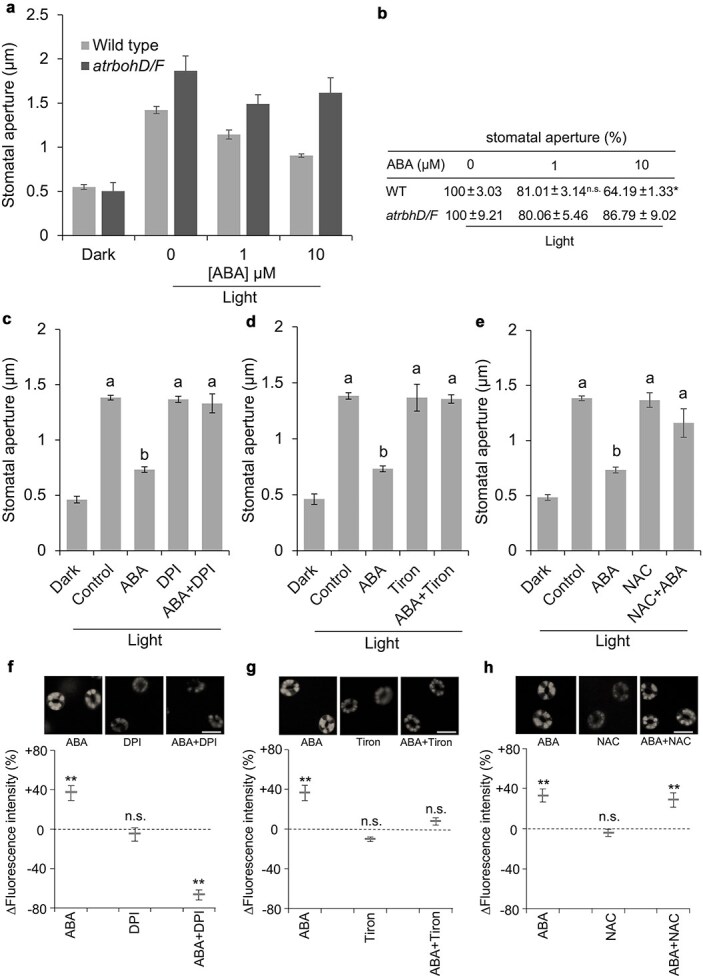
Involvement of ROS generation in inhibition of light-induced stomatal opening. (a) ABA inhibition of stomatal opening in the wild type and *rbohD rbohF* double mutant (*atrbohD*/*F*). (b) Aperture widths are indicated as % normalized to that of the solvent control (0.1% ethanol, 0 μM ABA). Asterisks indicate a significant difference between wild type and *atrbohD*/*F* mutant at 5% significance level by Student’s *t*-test. (c–e) effects of DPI, tiron, and NAC, respectively. Same letters indicate not significant at 5% level by Tukey’s honestly significant difference test. Wild-type plants were used in panels (C to H) DPI (12.5 μM), tiron (5 mM), and NAC (1 mM) were added at the same time as the addition of 10 μM ABA in the dark. Averages from five independent experiments (20 stomata per experiment) are shown. (f) Effect of DPI on ABA-induced H_2_O_2_ production, (g) effect of tiron on ABA-induced H_2_O_2_ production, (h) effect of NAC on ABA-induced H_2_O_2_ production. Representative grayscale H_2_DCF fluorescence images are shown in the top (F, G, and H) (scale bar = 10 μm). The vertical scale represents the percentage of H_2_DCF fluorescence intensity difference in the bottom panels. Fluorescent intensities are normalized to the control value taken as 100% for each experiment. Data are shown as mean ± SE of the mean (*n* = 3 independent experiments, 50 guard cells per experiment). Double asterisk indicates a significant difference at 1% significance level by Student’s *t*-test from each control. n.s., not significant.

We next investigated the involvement of ROS in inhibition of light-induced stomatal opening. A defect in opening inhibition was observed in *atrbohD/F* mutant (Fig. 5a and b and Supplementary Fig. S2b). Treatment with DPI, tiron, and NAC abolished the inhibition of stomatal opening by 10 μM ABA (Fig. 5c, d, and  e). These results indicate that the inhibition of light-induced stomatal opening is mediated by O_2_^−^• production by NADPH oxidase and successive formation of OH•. This is somewhat contradictory to our previous statement, which was based on the observation of a lack of H_2_O_2_ accumulation in the cytosol using the ROS indicator, H_2_DCF (Yin et al. 2013).

To comprehend this discrepancy in the involvement of ROS in the opening inhibition process, the effects of DPI, tiron, and NAC on H_2_DCF fluorescence that mainly detect H_2_O_2_ accumulation in the cytosol were examined. Pretreatment with DPI and tiron resulted in the inhibition of the increase in H_2_DCF fluorescence (Fig. 5f and g). On the contrary, NAC did not inhibit the H_2_O_2_ production (Fig. 5h). This difference can be explained by the difference in the reactivity of NAC against O_2_^−^• and OH•. In addition, pyrabactin did not induce H_2_O_2_ production in *atrbohD/F* (Supplementary Fig. S2c). Taken together with the previous study (Jannat et al. 2011), the results strongly support that inducible H_2_O_2_ accumulation in the cytosol mediates the induction of stomatal closure, and the NAC-sensitive OH• production plays an indispensable role in the inhibition of stomatal opening.

### Pyrabactin-mediated inhibition of seed germination and early seedling development

Seed germination and following early seedling development are essential for plant survival and propagation. ABA is known to regulate these processes (Finkelstein et al. 2002). However, mutations of ABA receptors result in great insensitivity to ABA. For instance, the *pyr1 pyl1 pyl2 pyl4* quadruple mutant and *pyr1 pyl1 pyl2 pyl4 pyl5 pyl8* sextuple mutant exhibit strong insensitivity to ABA inhibition of germination (Park et al. 2009, Gonzalez-Guzman et al. 2012). Here, we examined the effect of pyrabactin on seed germination and early seedling development.

Regarding radicle emergence, wild-type seeds showed a gradual increase in sensitivity to pyrabactin. In contrast, the sextuple mutant was markedly less sensitive, and although the quadruple mutant displayed a slight increase in sensitivity, its overall response was also significantly reduced. This pattern was consistent with the ABA response (Supplementary Fig. S3a and b).

In terms of cotyledon greening, both ABA and pyrabactin completely inhibited the wild type, while the sextuple mutant demonstrated reduced sensitivity. Its response was comparable to the quadruple mutant.

These findings suggest that PYR1, PYL1, PYL2, PYL4, PYL5, and PYL8 may function collectively as positive regulators of ABA signaling during seed germination and early seedling development. However, in contrast to the differential roles observed in guard cells, this functional divergence was not evident in response to pyrabactin during germination.

## Discussion

### Differential actions of ABA receptor isoforms in stomatal movements

More than 10 ABA receptor isoforms exist in plant genomes in general (e.g. 14 isoforms in the genome of *Arabidopsis thaliana*). Compared to other plant hormone receptors (such as, in the genome of *A. thaliana*, auxin 6 members, cytokinin 3 members, ethylene 5 members, GA 3 members, brassinosteroids 1 primary member, jasmonic acid 1 primary member, salicylic acid 3 members, strigolactones, 1 primary member), the number of ABA receptors is relatively large (Ma et al. 2009, Park et al. 2009, Raghavendra et al. 2010). The use of ABA agonists that bind only to a subset of the receptors may allow dissection of the complexity of ABA signaling. In this study, we examined the effects of pyrabactin on stomatal movements, second messenger mobilization and ion transporter activities in quadruple and sextuple mutants to explore the distinct combinations of ABA receptors involved in ABA signaling in guard cells. Particularly between the ABA action to induce stomatal closure and to inhibit stomatal opening. This system-level approach overcomes the potential confounding effects arising from pleiotropic influences and developmental compensation that may have been introduced through genetic mutant analysis in our previous study (Yin et al. 2013). Furthermore, it enables real-time analysis of stomatal responses within intact guard cells.

We found that pyrabactin-induced stomatal closure similarly to ABA (Fig. 1a and b), whereas it did not inhibit stomatal opening, unlike ABA (Fig. 1c and d). This biochemical evidence clearly showed that two distinctive behaviors of stomata, opening inhibition and closure induction, are governed at least partially by distinct ABA receptor isoforms, as hypothesized by earlier physiological studies (Anderson et al. 1994, Schwartz et al. 1994, Assmann and Jegla 2016). Inactivation of *I*_Kin_ was not undertaken by pyrabactin-recognizing ABA receptors (Fig. 2). Contrastingly, inactivation of H^+^-ATPase was undertaken by the pyrabactin-recognizing ABA receptors (Fig. 3). This suggests that stomatal aperture regulation is carried out by a combination of signaling pathways cued under distinct ABA receptor isoforms. The upregulation of ROS and NO levels, along with cytosolic alkalization was facilitated by the signaling events downstream of the ABA receptors that recognize pyrabactin (Fig. 4). Meanwhile, the downregulation of these signaling events seemed to be regulated in a subtly different manner from the upregulation. The difference in downregulation of these second messengers was detectable only in the mutant backgrounds (Fig. 4). This observation suggests that pyrabactin may function as a pharmacological antagonist for specific receptor subtypes. Moreover, it may function as an agonist, with distinct ABA receptor subtypes being involved in the processes of upregulation and the successive return to the resting state. The differential response to pyrabactin reflects variations in receptor subtype participation. The mechanism underlying pyrabactin’s effect on signal downregulation in the quadruple and sextuple ABA receptor mutants for the downregulation remains to be further elucidated. Differences in ABA signaling events regulated under a variety of ABA receptors in guard cells may give a clue for selective regulation of ABA responses (Vaidya et al. 2019), such as the control of plant transpiration rate, CO_2_ uptake, air pollutant entry, and pathogen invasions through distinct ABA receptor isoforms. This also offers the potential to selectively control stomatal behavior, which can enhance plant water use efficiency under abiotic stress conditions.

### Involvement of ROS generation in stomatal movement

It is well recognized that ROS plays a crucial role in ABA-induced stomatal closure (Pei et al. 2000, Zhang et al., 2001b, Kwak et al. 2003, Sirichandra et al. 2009). The ROS production in guard cells is discussed to be spatio-temporarily discriminated (Jannat et al. 2011, Gahir et al. 2024). Jannat et al. (2011) proposed that constitutive and inducible ROS elevations play different roles in guard cell signaling. NO production is anticipated to be associated with ROS production in the process. The levels of inducible H_2_O_2_ accumulation and that of inducible NO accumulation were correlated (Bright et al. 2006, Yan et al. 2007, Jannat et al. 2020). It is plausible that H_2_O_2_ and NO produce peroxynitrite and successive formation of nitrated cyclic GMP and eventually provoke Ca^2+^ channel activation (Gayatri et al. 2013, Joudoi et al. 2013, Jannat et al. 2020). Ca^2+^ elevation/oscillation occurring downstream of ROS production in the cytosol is likely a hallmark of steady-state closure of stomatal aperture (Allen et al. 2001).

Contrary to the advance in understanding ROS roles in closure induction, the role of ROS in opening inhibition was less understood. Yan et al. (2007) reported that exogenous application of H_2_O_2_ inhibited stomatal opening and concurrently induced NO production. They also showed that NO scavengers/NOS inhibitor reversed the H_2_O_2_ inhibition of light-induced stomatal opening. This pharmacological study suggests the involvement of H_2_O_2_ and NO productions in opening inhibition. Yamauchi et al. (2019) demonstrated genetic evidence that plant peroxisome-specific autophagy, so-called pexophagy, plays a role in regulating basal ROS levels in guard cells. The elevated ROS levels in the *atg* mutant having a defect in pexophagy caused reduction of light-induced stomatal opening. On the other hand, Yin et al. (2013) showed that H_2_O_2_ accumulation in guard cell cytosol is not required for opening inhibition.

**Figure 6:**
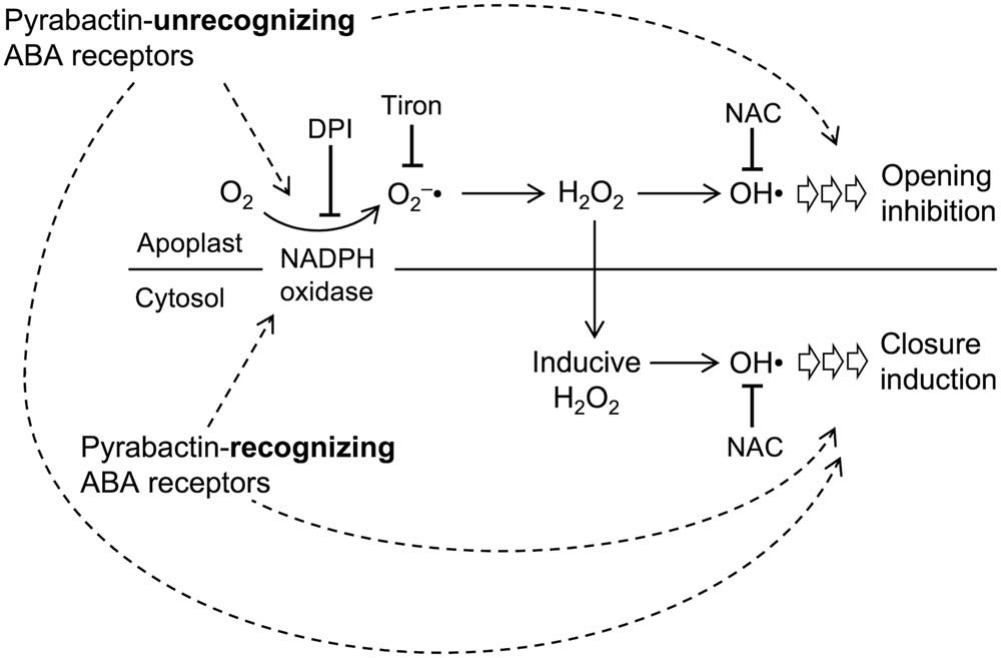
A proposed model shows differential actions of ROS in stomatal regulation. Pyrabactin-unrecognizing ABA receptors lead ROS production in the cytosol and the apoplast, inducing stomatal closure and inhibiting stomatal opening. Pyrabactin-recognizing ABA receptors only lead ROS production in the cytosol and induce stomata closure.

In this study, we examined the participation of ROS in the opening inhibition by pharmacological approaches using an NADPH oxidase inhibitor, DPI, and ROS scavengers, tiron and NAC; and a genetic approach using an NADPH oxidase double mutant, *atrbohD/F* (Fig. 5). It was shown that NADPH oxidase was unequivocally required for the ABA inhibition of stomatal opening. However, ABA did not induce H_2_O_2_ and NO accumulation in the cytosol of guard cells in quadruple and sextuple ABA receptor mutants. Meantime, light-induced stomatal opening was apparently inhibited in these mutants. Furthermore, in wild type, pyrabactin-induced accumulation of H_2_O_2_ and NO in the cytosol, while it did not inhibit stomatal opening. This discrepancy may be explained by the difference in responsive chemical species among ROS participating either in opening inhibition or closure induction. Tiron is a ROS scavenger that reacts readily with O2^−^• (Greenstock et al. 1975). On the other hand, NAC possesses negligible reactivity to H_2_O_2_ and O_2_^−^•, but a high reactivity to OH• (Aruoma et al. 1989, Aldini et al. 2018). ABA inhibition of stomatal opening was inhibited by both tiron and NAC (Fig. 5c and d). This indicates that the responsive ROS is OH• rather than O_2_^−^•. Looking into the effects of tiron and NAC on H_2_O_2_ accumulation in the cytosol of guard cells assessed by H_2_DCF fluorescence, we observed a difference: tiron inhibited H_2_O_2_ accumulation, while NAC did not (Fig. 5f and g). Tiron-sensitive O_2_^−^• production is a prerequisite for ABA-induced H_2_O_2_ accumulation in the cytosol of guard cells. In this process, NAC-sensitive OH• production may not be critical. Where is the place for tiron and NAC works, outside or inside the cell? These chemicals can occur both in the apoplast and the cytosol of guard cells, predicted by their octanol–water participation coefficients (Log*P* = −0.9 and 0.4, respectively, PubChem online database). Given that O_2_^−^• generated by NADPH oxidase and successively converted to H_2_O_2_ in the apoplast, followed by influx into cytosol across the plasma membrane; tiron blocks the O_2_^−^• production step in the apoplast. Cytosolic H_2_O_2_ accumulation brought about by this process (designated as inducible H_2_O_2_) is proposed to be involved in closure induction (Jannat et al. 2011). In the opening inhibition signal in guard cells, it is conceivable that an NAC-sensitive OH• production followed by the formation of H_2_O_2_ in the apoplast is a critical step (Fig. 6). Our findings provide that ABA response of stomatal is controlled by a combination of multiple ABA receptor isoforms that activate distinct fashions of ROS involvements in the downstream signaling network events.

## Conclusion

ABA induces stomatal closure and inhibits stomatal opening. Here, we successfully demonstrated that a synthetic ABA agonist, pyrabactin, controlled stomatal movement partly different from ABA: it induced stomatal closure and did not inhibit stomatal opening. Accordingly, the regulation of the plasma membrane *I*_Kin_ channel was not seen in the presence of pyrabactin, while the inhibition of H^+^-ATPase phosphorylation was observed. These findings offer new information on pyrabactin’s selectivity and demonstrate that the stomatal movement machinery is differently regulated by a combination of multiple ABA receptor isoforms. In addition, pharmacological analysis of ROS scavengers revealed the involvement of ROS in a different manner in closure induction and opening inhibition. We propose a new insight for regulating stomatal movements selectively, aiming to maximize plants’ water use efficiency.

## Materials and Methods

### Plant materials

*A. thaliana* (L.) Heynh. ecotype Columbia (Col-0), *pyr1 pyl1 pyl2 pyl4 pyl5 pyl8* sextuple mutant (Gonzalez-Guzman et al. 2012), *pyr1 pyl1 pyl2 pyl4* quadruple mutant (Park et al. 2009) and *atrbohD/F* double mutant (Kwak et al. 2003) were grown on a soil mixture consisting of 70% (v/v) vermiculite (Asahi-kogyo) and 30% (v/v) Kureha soil (Kureha Chemical). Temperature and RH in the growth chamber were controlled at 22°C ± 2°C and 60% ± 10%, respectively. The photoperiod was a 16-h-light/8-h-dark regime, with a photon flux at 80 μmol m^−2^ s^−1^ with white fluorescent tubes.

### Measurement of stomatal aperture

Closure induction and opening inhibition of the stomatal aperture were measured as previously described (Munemasa et al. 2019). In brief, or closure induction assay, an excised rosette leaf was floated on the opening buffer containing 5 mM KCl, 50 μM CaCl_2_, and 10 mM MES-Tris (pH 6.15) for 2 h in the light (80 μmol m^−2^ s^−1^) to pre-open stomata. Then, pyrabactin (product number B-3538, Sigma-Aldrich Inc., St. Louis, MO, USA) or ABA (Sigma-Aldrich Inc.) was added to the opening buffer and further incubated for 2 h in the light. Aperture width was measured under a microscope after the incubation following the release of epidermal specimen by blending for 30 s with a Waring commercial blender (BB-700, Waring Products, Torrington, MO, USA), unless otherwise stated.

For opening inhibition assay, a rosette leaf excised from a dark-adapted plant (overnight in the dark for 8 h) was blended with a blender under a dim green light (without blue and red light) before the onset of the light period. The released epidermal specimens were suspended in the opening buffer in the dark. After a subsequent 2-h incubation in the dark, they were incubated in the light for 2.5 h in the presence or absence of pyrabactin or ABA. Aperture width was measured under a microscope.

### Measurement of H_2_O_2_ and NO production

Production of H_2_O_2_ and NO in guard cells was examined using H_2_DCF-DA (Sigma-Aldrich Inc.) and DAF-2DA (Sigma-Aldrich Inc.), respectively, as described elsewhere (Yin et al. 2016, Munemasa et al. 2019). In brief, epidermal peels were collected from leaves of 4- to 6-week-old plants by blending, incubated for 2 h in the opening buffer in the light, and incubated with 50 μM H_2_DCF-DA or 5 μM DAF-2DA for 30 min at room temperature to load the dye into the guard cells, followed by rinsing with distilled water on a nylon mesh to remove excess dye. Dye-loaded epidermis was successively treated with 10 μM pyrabactin or ABA (or solvent control) for 20 min. Fluorescence in guard cells was imaged using a fluorescence microscope (BZ-8000, Keyence Corporation, Osaka, Japan). Fluorescence intensity in a guard cell was semiquantified using ImageJ software (http://imagej.net).

### Measurement of cytosolic pH change

Change in cytosolic pH (pH_cyt_) in guard cells was examined using BCECF-AM (Dojindo, Kumamoto, Japan) according to Yin et al. (2016). Epidermal tissues isolated from 4- to 6-week-old plants by blending were incubated for 3 h under light (80 μmol m^−2^ s^−1^) in the opening buffer. The epidermises were then incubated with 20 μM BCECF-AM in the opening buffer in the dark at room temperature for 30 min to load the dye. Loading was terminated by rinsing the tissues three times with the opening buffer. Ten μM pyrabactin or ABA (or solvent control) was added and incubated for 20 min under the light. Fluorescence images of guard cells were captured with a fluorescence microscope (BZ-8000, Keyence Corporation, Osaka, Japan). Fluorescence intensity in guard cells was semiquantified using ImageJ.

### Patch-clamp measurement

Inwardly rectified K^+^ current (*I*_kin_) was measured by whole-cell patch clamping of isolated guard cell protoplasts according to the previous report (Yin et al. 2016).

### Phosphorylation of plasma membrane H^+^-ATPase in guard cells

Blue light-induced phosphorylation of the plasma membrane H^+^-ATPases in guard cells was examined immunohistochemically using an antiserum against the phosphorylated penultimate threonine of H^+^-ATPase polypeptide (anti-pThr) according to the previous report (Hayashi et al. 2011). In brief, epidermal tissues were isolated from the leaf of dark-adapted plants with a commercial blender in the dark and incubated under background red light at 50 μmol m^−2^ s^−1^ for 20 min in the incubation buffer containing 5 mM KCl, 50 μM CaCl_2_, and 10 mM MES-Tris (pH 6.15) with 10 μM pyrabactin or ABA (or solvent control), followed by a 2.5-min at 10 μmol m^−2^ s^−1^ blue light treatment. The epidermal tissues were fixed at before and after blue light irradiation. After these treatments, the epidermal fragments were collected on a sheet of nylon net. The collected fragments were fixed with paraformaldehyde and stuck on a coverslip, followed by a treatment with 3% driselase 20 (Kyowa Hakko Kogyo Co., Ltd., Tokyo) and 0.5% macerozyme R-10 (Yakult Pharmaceutical Industry Co., Ltd., Tokyo) for 45 min at 37°C. After permeabilizing the cells with 3% Triton X-100 for 30 min at room temperature, phosphorylated H^+^-ATPases were visualized with anti-pThr at a dilution of 1:1000 in phosphate-buffered saline (PBS) with 3% BSA and an Alexa Fluor488-labeled secondary antibody at a dilution of 1:500 in PBS with 3% BSA as described in Hayashi et al. (2011). The fluorescent images were observed with a fluorescence microscope (BX50, Olympus) equipped with a light-emitting diode and LDP light source (U-LGPS, Olympus) and captured using a CCD camera system (DP71, Olympus). The intensity of fluorescence was quantified with ImageJ.

### Germination and seedling growth assays

Sterilized seeds were sown on 1/2-strength MS medium supplemented with 0, 0.1, 0.5, and 1 μM ABA or pyrabactin, incubated in the dark at 4°C for 3 days, and then transferred to a growth chamber. Seeds were considered germinated when their radicles protruded from the testa, and seedling growth was confirmed by the development of green expanded cotyledons. The percentages of radicle emergence and cotyledon greening were recorded at indicated time points.

### Statistical analysis

Student’s *t*-test and ANOVA with Tukey–Kramer *post hoc* test or Dunnett’s *post hoc* test were used to assess the significance of differences between datasets. The difference at the level of *P* < .05 was regarded as significant.

## Supplementary Material

pcp-2024-e-00272-File008_pcaf102

## Data Availability

Sequence data used in this article can be found in the Arabidopsis Information Resource database (https://www.arabidopsis.org/) under the following accession numbers: PYR1 (At4g17870), PYL1 (At5g46790), PYL2 (At2g-26040), PYL4 (At2g38310), PYL5 (At5g05440), PYL8 (At5g53160), AtrbohD (At5g47910), and AtrbohF (At1g64060). The authors confirm that the data supporting the findings of this study are available within the article and its online supplementary materials.
