# Supplementary material for: ABA receptor isoforms differently regulate stomatal movements and generation of reactive oxygen species in ABA signaling in Arabidopsis guard cells
Source: Plant Cell Physiol. 2025 Aug 28;66(12):1811–22. doi: 10.1093/pcp/pcaf102 (PMC12739107; doi:10.1093/pcp/pcaf102)
Supplement: pcp-2024-e-00272-File008_pcaf102 [file pcp-2024-e-00272-file008_pcaf102.docx]

Title: ABA Receptor Isoforms Differently Regulate Stomatal Movements and Generation of Reactive Oxygen Species in ABA Signaling in Arabidopsis Guard Cells

Running Title: Regulation of stomata by ABA receptor isoforms

Ye Yin^1,2^, Yuki Hayashi^3^, Monira Sirajam^1^, Oumayma Shaiek^1^ Shintaro Munemasa^1^, Yoshimasa Nakamura^1^, Toshinori Kinoshita^3,4^, Yoshiyuki Murata^1^, Izumi C. Mori^5,^*

^1^ Graduate School of Environmental and Life Science, Okayama University, Kita-ku, Okayama, 700–8530 Japan

^2^ College of Horticulture, Qingdao Agricultural University, Qingdao, 266109, China

^3^ Graduate School of Science, Nagoya University, Chikusa, Nagoya, 464-8602 Japan

^4^Institute of Transformative Bio-Molecules (WPI-ITbM), Nagoya University, Chikusa, Nagoya, 464-8602 Japan

^5^Institute of Plant Science and Resources, Okayama University, Kurashiki, Okayama, 710–0046, Japan

*Corresponding Author: Email: [imori@okayama-u.ac.jp](mailto:imori@okayama-u.ac.jp); Fax: 086-434-1249

**Table S1** Effect of ABA on stomatal closure in *pyr1 pyl1 pyl2 pyl4* quadruple mutant guard cells.

a, Independent experiment (20 stomata were measured per experiment); b, values of Dunnett’s test compared to the 0 µM ABA control (Published in Yin et al. (2013).

**Table S2**. Effect of 10 µM ABA on ROS and NO production and cytosolic pH in *pyr1 pyl1 pyl2 pyl4* quadruple mutant guard cells.

a, Independent experiment (20 stomata were measured per experiment); b, P values of Student’s *t*-test compared to the 0 µM ABA control (Published in Yin et al. (2013).

**Figure S1.** Involvement of ROS generation in ABA-induced stomatal closure. (A) ABA-induced stomatal closure in wild type and *rbohD rbohF* double mutant (*atrbohD/F*). Asterisks indicate significant differences between wild type and *atrbohD/F* at 5% significance level (*) or 1% significance level (**) by Student’s *t*-test. (B) to (D) Effects of diphenyleneiodonium (DPI), tiron, and N-acetylcysteine (NAC) on ABA-induced stomatal closure. Same letters indicate not significant at 5% level by Tukey’s honestly significant difference test. Wild-type plants were used in panels B to D. DPI (12.5 µM), tiron (5 mM) and NAC (1 mM) were added at the same time as the addition of 10 µM ABA. Averages from five independent experiments (20 stomata per experiment) are shown. Error bars represent standard error of the mean.

**Figure S2.** Effects of pyrabactin on stomatal movements and H_2_O_2_ accumulation levels in the wild type and *rbohD rbohF* double mutant (*atrbohD/F*) guard cells. (A) Closure induction assay. (B) Opening inhibition assay. Averages from five independent experiments (20 stomata per experiment) are shown. (C) Effect of ABA (10 μM) and pyrabactin (10 μM) on cytosolic H_2_O_2_ level. The vertical scale in panel C represents the percentage difference in H_2_DCF fluorescence intensity in guard cells. Data are shown as mean ± standard error of the mean (n = 3 independent experiments, 50 guard cells per experiment). Double asterisk indicates significant difference at 1% by Student’s t-test from the control. n.s. indicates not significant.

**Figure S3.**The effect of ABA and pyrabactin on seed germination. (A and B) radicle emergence rate, (C, D) Cotyledon greening rate were determined 2–5 days after stratification on 1/2-strength MS medium without or with 0.1 μM, 0.5 μM, 1 μM ABA or pyrabactin. Averages from three independent experiments are shown (10 seeds/genotype/repeat). Same letters indicate not significant at 5% level by Tukey’s honestly significant difference test. Error bars represent standard error of the mean.
